# Whole Genome Analysis Detects the Emergence of a Single *Salmonella enterica* Serovar Chester Clone in Japan’s Kanto Region

**DOI:** 10.3389/fmicb.2021.705679

**Published:** 2021-07-27

**Authors:** Naoshi Ando, Tsuyoshi Sekizuka, Eiji Yokoyama, Yoshiyuki Aihara, Noriko Konishi, Yuko Matsumoto, Kumiko Ishida, Koo Nagasawa, Nathalie Jourdan-Da Silva, Motoi Suzuki, Hirokazu Kimura, Simon Le Hello, Koichi Murakami, Makoto Kuroda, Shinichiro Hirai, Setsuko Fukaya

**Affiliations:** ^1^Division of Bacteriology, Chiba Prefectural Institute of Public Health, Chiba, Japan; ^2^Pathogen Genomics Center, National Institute of Infectious Diseases, Tokyo, Japan; ^3^Division of Bacteriology, Ibaraki Prefectural Institute of Public Health, Mito, Japan; ^4^Department of Microbiology, Tokyo Metropolitan Institute of Public Health, Tokyo, Japan; ^5^Microbiological Testing and Research Division, Yokohama City Institute of Public Health, Yokohama, Japan; ^6^Itako Public Health Center, Itako, Japan; ^7^Laboratory of Cancer Genetics, Chiba Cancer Center Research Institute, Chiba, Japan; ^8^Santé Publique France, The French National Public Health Agency, Saint-Maurice, France; ^9^Center for Surveillance, Immunization, and Epidemiologic Research, National Institute of Infectious Diseases, Tokyo, Japan; ^10^Faculty of Health Science, School of Medical Technology, Gunma Paz University, Takasaki, Japan; ^11^French National Reference Center for E. coli, Shigella and Salmonella, Institute Pasteur, Paris, France; ^12^Groupe de Recherche sur l’Adaptation Microbienne (GRAM 2.0, EA2656), Normandy University, UNICAEN, UNIROUEN, Caen, France; ^13^Center for Emergency Preparedness and Response, National Institute of Infectious Diseases, Musashi-Murayama, Japan

**Keywords:** *Salmonella* Chester, emerging, molecular epidemiological analysis, population genetic analysis, whole genome sequence data, single-nucleotide polymorphisms, selection pressure

## Abstract

In Japan’s Kanto region, the number of *Salmonella enterica* serovar Chester infections increased temporarily between 2014 and 2016. Concurrently with this temporal increase in the Kanto region, *S*. Chester isolates belonging to one clonal group were causing repetitive outbreaks in Europe. A recent study reported that the European outbreaks were associated with travelers who had been exposed to contaminated food in Morocco, possibly seafood. Because Japan imports a large amount of seafood from Morocco, we aimed to establish whether the temporal increase in *S*. Chester infections in the Kanto region was associated with imported Moroccan seafood. Short sequence reads from the whole-genome sequencing of 47 *S*. Chester isolates from people in the Kanto region (2014–2016), and the additional genome sequences from 58 isolates from the European outbreaks, were analyzed. The reads were compared with the complete genome sequence from a *S*. Chester reference strain, and 347 single nucleotide polymorphisms (SNPs) were identified. These SNPs were used in this study. Cluster and Bayesian cluster analyses showed that the Japanese and European isolates fell into two different clusters. Therefore, *Φ_*PT*_* and $I_{A}^{S}$ values were calculated to evaluate genetic differences between these clusters. The results revealed that the Japanese and European isolates were genetically distinct populations. Our root-to-tip analysis showed that the Japanese isolates originating from one clone had accumulated mutations, suggesting that an emergence of this organism occurred. A minimum spanning tree analysis demonstrated no correlation between genetic and geographical distances in the Japanese isolates, suggesting that the emergence of the serovar in the Kanto region did not involve person-to-person contact; rather, it occurred through food consumption. The *d*_*N*_/*d*_*S*_ ratio indicated that the Japanese strain has evolved under positive selection pressure. Generally, a population of bacterial clones in a reservoir faces negative selection pressure. Therefore, the Japanese strain must have existed outside of any reservoir during its emergence. In conclusion, *S*. Chester isolates originating from one clone probably emerged in the Kanto region via the consumption of contaminated foods other than imported Moroccan seafood. The emerging strain may have not established a reservoir for survival in the food supply chain resulting in its disappearance after 2017.

## Introduction

The number of *Salmonella enterica* serovar Chester infections increased temporarily during 2014–2016 in the Kanto region of Japan. We reported that an outbreak in 2016 from an unknown infection source was caused by *S. enterica* subsp. *enterica* serovar Chester in a part of the aforementioned Japanese region (Aoki et al., 2020). In this outbreak, seven *S*. Chester infection cases occurred in the adjacent prefectures of Chiba and Ibaraki in October and November of 2016. No epidemiological link among the cases except for an intrafamily case was found. Simultaneously, pulsed-field gel electrophoresis (PFGE) showed that all 10 of the isolates had almost identical band patterns (Aoki et al., 2020), which raises the possibility that these isolates originated from one clone. Non-typhoidal *Salmonella* (NTS) is a significant cause of foodborne illness worldwide (World Health Organization, 2018)^1^. In Japan, infections with *S*. Chester have been rare among all reported NTS infections (Katoh et al., 2015). Nevertheless, the number of *S*. Chester isolates in Tokyo, a part of the Kanto region, increased temporarily in 2014–2016 (Tokyo Metropolitan Infectious Disease Surveillance Center, 2020)^2^, while they did also in other areas of Kanto region, that is, Chiba Prefecture, Ibaraki Prefecture and Yokohama City. In the period, 44 *S*. Chester isolates were isolated in the Kanto region, which included nine isolates from the seven infection cases that we reported previously (Aoki et al., 2020), although *S*. Chester was not isolated before or after this period. Previously, the number of *S*. Oranienburg isolates, another rarely isolated serovar in Japan, increased in various areas of Japan in 1998–1999; subsequently, PFGE analysis demonstrated that the temporary increase was caused by a diffuse outbreak (Tsuji and Hamada, 1999). Therefore, the *S*. Chester isolates that emerged during 2014–2016 might belong to a single diffuse outbreak and include the outbreak isolates from 2016 (Aoki et al., 2020) in the Kanto region.

Looking outside of Japan, an interesting fact has emerged. In Europe, *S*. Chester isolates belonging to one clonal group have repetitively caused outbreaks in recent years (Fonteneau et al., 2017). In the summers of 2014 and 2015, a multinational outbreak caused by this bacterium occurred across several countries (e.g., France, Belgium, Netherlands, Spain, Denmark, and Sweden). From April to October in 2016, the number of *S*. Chester infections increased in France. Fonteneau et al. (2017) reported that most of the *S*. Chester cases had traveled to Morocco and eaten seafood there before the onset of symptoms. This implies that the *S*. Chester isolates belonging to the same clonal group had spread continuously in Morocco and that the spreading isolates have caused outbreaks every year in Europe, perhaps via the consumption of Moroccan seafood. As for Japan, a large amount of seafood (e.g., octopus, squid, and eel) has been imported from Morocco (Ministry of Agriculture, Forestry and Fisheries, 2019)^3^. Some cases of *Salmonella* spp. infections have previously been caused by seafood and fish consumption (Tsuji and Hamada, 1999; Heinitz et al., 2000). Therefore, we hypothesize that imported seafood from Morocco was one of the vehicle candidates for *S*. Chester infections in the Kanto region of Japan during 2014–2016; that is, the temporary increase in the infections in the Kanto region might be caused by the *S*. Chester isolates spreading in Morocco.

Several studies have recently shown the superiority of whole genome sequencing (WGS) data compared with PFGE data. PFGE has generally been used to determine whether bacterial isolates originate from the same outbreak. However, PFGE can only analyze nucleotide differences between isolates at a relatively small number of restriction enzyme sites (Kudva et al., 2004). Therefore, PFGE has sometimes failed to differentiate isolates originating from the same outbreak, that is, isolates derived from one clone, and isolates originating outside the outbreak. During 2014–2015, enterohemorrhagic *Escherichia coli* (EHEC) O121 isolates with similar PFGE patterns were isolated in various Japanese regions. Analysis of single nucleotide polymorphism (SNP) extracted from WGS data showed that only some of EHEC O121 isolates were derived from a single clone, while the WGS data showed that the remaining isolates were not from this clone (Lee et al., 2017). The superiority of WGS data analysis for molecular epidemiological analysis was also apparent in the cases of EHEC O157 (Yokoyama et al., 2018) and *S*. Agona (Yokoyama et al., 2019).

Therefore, the aim of this study was to clarify whether the temporal increase in *S*. Chester infections in the Kanto region of Japan was associated with imported seafood consumption from Morocco. First, we performed molecular epidemiological and population genetic analyses using WGS data to elucidate the relationship between *S*. Chester isolates from the Kanto region and those from Europe. Second, we investigated whether a correlation exists between the genetic and geographical distances of the Japanese isolates using a molecular epidemiological analysis to confirm that the Kanto region’s temporal increase occurred via contaminated food. Third, our WGS data was used to assess whether selective pressure featured in the outbreaks involving *S*. Chester. We then discuss why the *S*. Chester isolates emerging in the Kanto region during 2014–2016 were not isolated after 2017.

## Materials and Methods

### Collection of *S*. Chester Isolates Isolated in the Kanto Region of Japan

*Salmonella* Chester isolates isolated in the Kanto region (47 isolates) during 2014–2016 were used in this study (Supplementary Table 1 and Supplementary Figure 1). A traditional contact survey was carried out by local health centers in each municipality. When a local public health institute in Japan receives a report that a food poisoning or infection case has occurred, the institute microbiologically examines the pathogens to identify which one(s) is/are causing the case. For this study, we made inquiries to all local public health institutes in the Kanto region as to whether the number of *S*. Chester isolates derived from human and food sources had increased recently. On the inquiry, it became clear that the number of *S*. Chester isolates from humans increased in Tokyo Metropolitan, Chiba Prefecture, Ibaraki Prefecture and Yokohama City during 2014–2016. However, *S*. Chester isolates were not isolated by these institutes before or after this period. In this study, nine *S*. Chester isolates from people in Tokyo, 18 from Chiba, 11 from Ibaraki, and nine from Yokohama were collected (Supplementary Table 1).

### SNP Detection From WGS Data

The short sequence reads from 58 European *S*. Chester isolates (ST1954) that were collected from humans, chickens and food in the multinational European outbreaks were obtained in a previous study (Fonteneau et al., 2017). These reads are deposited in the National Center for Biotechnology Information Short Read Archive (BioProject PRJNA248792). The short sequence reads from the European isolates were kindly provided by the Institute Pasteur (France) to the National Institute of Infectious Diseases (NIID), Japan.

As for the Japanese isolates, the short sequence reads from the 47 *S*. Chester isolates from the Kanto region of Japan were obtained by the NIID. DNA was extracted from the Japanese isolates and purified using the Isoplant II DNA extraction kit (Nippon Gene Inc., Tokyo, Japan). The genomic DNA libraries were prepared using the Nextera XT DNA sample prep kit (Illumina). The pooled libraries were subjected to multiplexed paired-end sequencing (300 mer × 2) using MiSeq (Illumina, San Diego, CA, United States). The adapter sequences were trimmed from the short reads, and low quality bases with Phred scores less than 15 were eliminated using the Skewer program (Jiang et al., 2014) to obtain sequences of at least 50-mers in length. In the NIID, the short reads from the Japanese and European isolates were mapped using the BWA-mem program (Li and Durbin, 2010) against the *S*. Chester ATCC 11997 strain’s complete genome sequence (4,660,922 bp, GenBank accession number CP019178.1) as a reference (Supplementary Table 2). After excluding prophage, repeat, indel, biallelic, and recombination regions from the complete genome sequence, 91.122% (4,293,757 bp) of the genome was used for variant detection. Variant calling was performed using VarScan ver. 2.3.4 (Koboldt et al., 2009), resulting in the identification of 347 SNPs (Supplementary Table 3). Out of the 347 SNPs, 120 and 271 SNPs were detected in the Japanese and European isolates, respectively. Forty-four SNPs were common to both the Japanese and European isolates. The 347 SNPs were used for further analyses in this study.

### Molecular Epidemiology

To perform multilocus sequence typing (MLST) on the Japanese isolates, the assembled sequence contigs from the A5-MiSeq pipeline (Coil et al., 2015) were analyzed using the web tools available from the Center for Genomic Epidemiology website^4^ (Larsen et al., 2012). The MLST classified the *Salmonella* isolates into sequence types (STs) based on the analysis of seven housekeeping genes (i.e., *aroC*, *dnaN*, *hemD*, *hisD*, *purE*, *sucA*, and *thrA*) (Achtman et al., 2012). The chromosomal positions of these housekeeping genes in the *S*. Chester ATCC 11997 strain (Accession number CP019178.1) were 1,427,763–1,428,848 for *aroC*, 1,406–2,506 for *dnaN*, 4,565,282–4,566,022 for *hemD*, 1,788,720–1,790,024 for *hisD*, 1,788,720–1,790,024 for *purE*, and 3,104,010–3,106,100 for *sucA*, and 3,872,960–3,873,009 for *thrA*. The European isolates were classified as belonging to ST1954 based on the MLST analysis in our previous study (Fonteneau et al., 2017).

Cluster analysis was conducted using MEGA 7 software (Kumar et al., 2016). The 347 SNPs from the Japanese and European isolates were imported into the software, and a dendrogram was reconstructed by the maximum-likelihood method with the Tamura-Nei model and with 1,000 bootstraps performed for the statistics. The other conditions for reconstructing the dendrogram were set by the default settings.

Bayesian cluster analysis using the 347 SNPs from the Japanese and European isolates was conducted using STRUCTURE Version 2.3.4 software (Pritchard et al., 2000). The setting options were as follows: length of burn-in period was 50,000, the Markov Chain Monte Carlo number was 100,000, the ancestry model was the “admixture model,” and the frequency model was “allele frequency correlated.” To determine the number of clusters (*K*) to show how the isolates could be grouped, delta *K* was calculated using STRUCTURE Harvester Ver. 0.6.93 software (Earl and von Holdt, 2012).

### Population Genetic Analysis

The difference between the Japanese and European isolates was evaluated using *Φ_*PT*_*, which is an analogue of *Fst* (Peakall and Smouse, 2012). Genetic Analysis in Excel (GenAlEx), version 6.51b2 software, an add-on package from Microsoft Excel, was used to calculate a *Φ_*P*_*_*T*_ value with 999 permutations. *Φ_*PT*_* was calculated as the proportion of the variance among populations from:

$$\Phi_{\mathrm{PT}}=\frac{V_{\mathrm{AP}}}{V_{\mathrm{AP}}+V_{\mathrm{WP}}}$$

Where *V_*AP*_* was the variance among the populations and *V*_*WP*_ was the variance within a population. If a *Φ_*PT*_* value differed significantly from zero, the Japanese and European isolates would be different populations.

Linkage disequilibrium in the Japanese and European isolates was evaluated using the standardized index of association ($I_{A}^{S}$) (Haubold and Hudson, 2000). LIAN Ver. 3.7 software was used to calculate $I_{A}^{S}$ for each of the Japanese and European isolates from the ratio of the variance of the observed mismatches in the test set (*V*_*D*_) to the variance expected for a state of linkage equilibrium (*V*_*e*_), scaled by the number of loci used in the analysis (*l*), from:

$$I_{A}^{S}=\frac{1}{l-1}\,\left(\frac{V_{D}}{{\text{V}}_{e}}-1\right)$$

The significance between *V*_*D*_ and *V*_*e*_ was determined by the Monte Carlo simulations with 10^3^ resamplings.

### Comparison of Genetic Traits Between the Japanese and European Isolates

A root-to-tip analysis was performed using Path-O-Gen software to evaluate whether changes occurred in the diversity of the Japanese and European isolates as time proceeds (Rambaut et al., 2016). In the root-to-tip analysis, the X-axis shows the isolation date for each isolate, and the Y-axis shows the genetic distance to each isolate from the root on the dendrogram. If the isolates originating from the same clone are assumed to have a uniform mutation rate, they would show a proportionate relationship between the X-and Y-axes.

The frequencies of the SNPs causing non-synonymous (*P*_*N*_) and synonymous (*P*_*S*_) substitutions in the amino acids were compared to evaluate the selection pressure on each of the Japanese and European isolates as follows (Bierne and Eyre-Walker, 2004). Of the 347 SNP loci detected in the WGS data, 300 and 47 of them were on coding and non-coding regions, respectively. Out of the 300 SNPs, there were two types of nucleotide bases in 66 of the SNPs in the sequences from the Japanese isolates and 210 from the European isolates. *L*_*N*_ and *L*_*s*_ represent the number of SNP loci causing non-synonymous and synonymous substitutions, respectively. We designated the minor allele to be the lesser allele belonging to the two alleles in a SNP locus. If two alleles in a SNP locus are equal, either allele was designated as the minor allele. *SL_*N*_ x* and *SL*_*S*_ *x* represents the number of Japanese and European isolates where the minor alleles at the *x*th SNP locus cause non-synonymous and synonymous substitutions, respectively. *N* is the number of Japanese and European isolates. *P*_*N*_ and *P*_*S*_ can be calculated from:

$$d_{N}=\left(\sum_{x=1}^{L_{N}}{\mathrm{SL}}_{N}\,x\right)\,/\,\left(L_{N}\times N\right)$$

$$d_{S}=\left(\sum_{x=1}^{L_{S}}{\mathrm{SL}}_{S}\,x\right)\,/\,\left(L_{S}\times N\right)$$

When the *d*_*N*_/ *d*_*S*_ ratios were >1 and <1, the genes possessed by each group were considered to have been subjected to positive or negative selection, respectively (Stukenbrock and Bataillon, 2012). Where *d*_*N*_/*d*_*S*_ = 1 this indicates that the group has evolved under neutral evolution.

A minimum spanning tree (MST) analysis was performed using PopART software Version 1.7 (Leigh and Bryant, 2015) to evaluate how the Japanese isolates emerged geographically as the time advanced. All the SNPs in the sequences from the Japanese isolates were imported into the software, and an MST was reconstructed using the default epsilon value (0).

The presence of SNPs in four genes (i.e., *mut*S, *mut*L, *mut*H, and *uvr*D) within the mismatch repair system (Sheng et al., 2020) was investigated. The chromosomal positions of these genes in the *S*. Chester strain ATCC 11997 (Accession number CP019178.1) were 956,162–958,729 for *mut*S, 4,121,661–4,123,517 for *mut*L, 3,712,806–3,713,201 for *mut*T, and 4,551,149–4,553,311 for *uvr*D. When at least one SNP was located in these genes, we examined whether the SNP caused either non-synonymous or synonymous substitutions.

### DNA Sequence Data Deposition

The short sequence reads from the 47 Japanese isolates have been deposited in the Sequence Read Archive in the DNA Data Bank of Japan (Supplementary Table 4).

## Results

### Discrimination of the Japanese and European Isolates

The MLST analysis showed that all the Japanese isolates belong to ST1954, which is the same sequence type as that of the European isolates (Fonteneau et al., 2017). In detail, the nucleotide sequences in each of the seven housekeeping genes (i.e., *aroC*, *dnaN*, *hemD*, *hisD*, *purE*, *sucA*, and *thrA*) were exactly the same among the Japanese and European isolates. This MLST result is consistent with no SNPs being detected in these seven genes among the Japanese and European isolates, as determined by mapping the short sequence reads from these isolates against the reference genome sequence (Supplementary Table 3).

**FIGURE 1:**
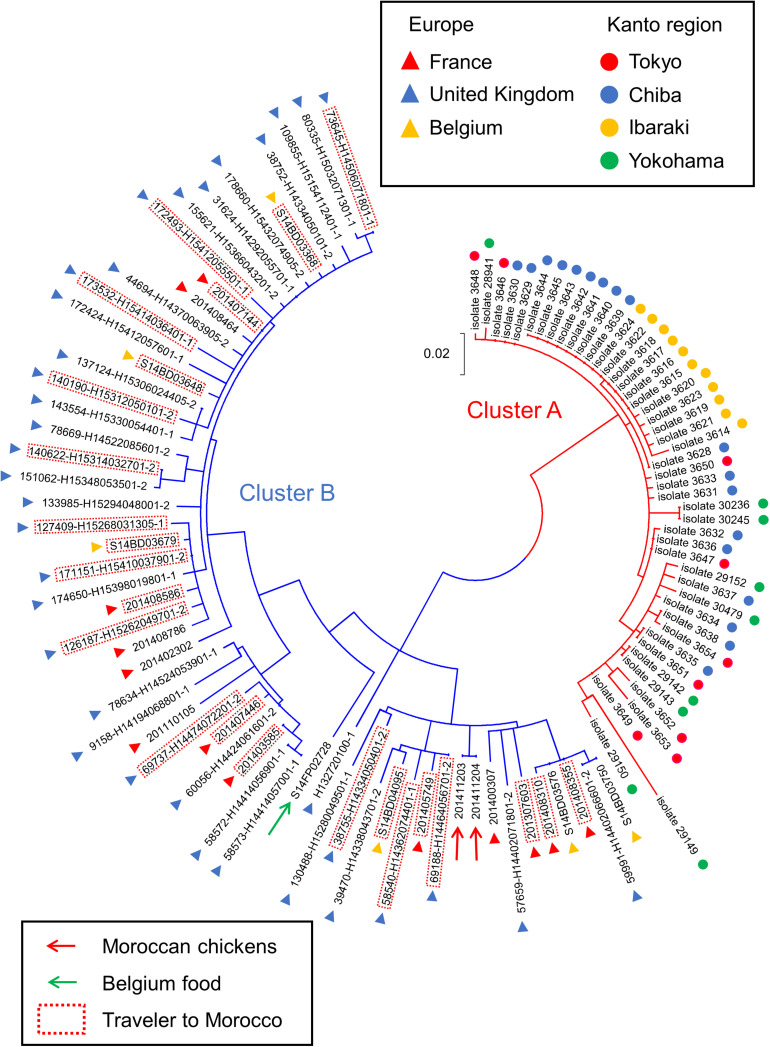
Dendrogram reconstructed from SNPs from *Salmonella* Chester isolated in the Kanto region of Japan and Europe. Branches for the isolates included in clusters A and B are indicated in red and blue, respectively. Countries and areas where the isolates were collected are indicated by colored circles and triangles. Isolates derived from travelers to Morocco are surrounded by red dotted lines. Red and green arrows show the isolates obtained from chickens or food, respectively. The scale bar indicates the genetic distance.

The cluster analysis showed that the Japanese and European isolates fell into two clusters (i.e., clusters A and B), one of which only contains the Japanese isolates (cluster A), while the other contains only the European isolates (cluster B) (Figure 1). We found that cluster B includes the *S*. Chester isolates from travelers to Morocco, one chicken isolate from Morocco, and two isolates derived from food in Belgium. In this dendrogram, there were no *S*. Chester isolates that were not included in cluster A or B. The genetic distances from the root to clusters A and B were 0.052 and 0.043, respectively. Therefore, the cut-off value for the genetic distance we used to define a cluster was determined to be 0.04 in this study. The maximum pair-wise genetic distance between the *S*. Chester isolates in cluster A (i.e., isolate 29149) and cluster B (i.e., 201110105) was 0.327 (Supplementary Table 5). The pair-wise comparison of the genetic distances between all 105 *S*. Chester isolates is shown in Supplementary Table 5.

**FIGURE 2:**
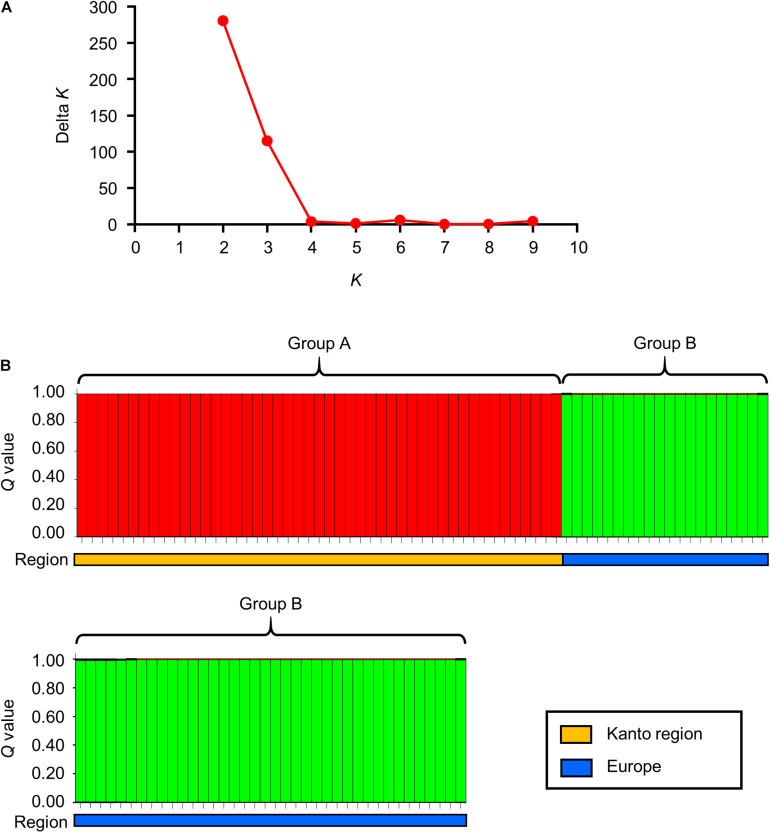
Bayesian cluster analysis of *S*. Chester isolated in the Kanto region of Japan and Europe. **(A)** This graph represents Delta *K* (Y-axis) against the assumed sub-populations (X-axis). **(B)** This panel shows the genetic structure of the 105 *S*. Chester isolates from the Kanto region and from Europe under the assumption of *K* = 2. **(B)** This panel contains 105 red and light green squares, and the height of each square indicates the posterior probability value (*Q*) of the isolate. These red and light green squares represent groups A and B, respectively. The orange and blue bars below the 105 squares indicate the region where each isolate originated (i.e., the Kanto region or Europe). The details of this analysis are described in Supplementary Table 1.

The division of the two clusters on the dendrogram was also confirmed by Bayesian cluster analysis. This analysis showed that the highest Delta *K* value was obtained when a population was partitioned in two sub-populations (Figure 2A). Among the 10 runs under the assumption of *K* = 2, the highest log likelihood value was obtained in the third run with ln *P*(*D*) = −3479.8. In the third run, when the isolates were ordered according to their posterior probability values (*Q*) (Supplementary Table 1), they formed two groups that were completely concordant with the two clusters on the dendrogram (Figure 2). The *Q* values of all the Japanese isolates in group A and the values of all the Europe isolates in group B were >0.99.

The division of the Japanese and European isolates into these two groups was supported by the *Φ_*PT*_* value. The value was 0.700 (*P* = 0.001), indicating that the two groups belonged to different populations. Furthermore, the $I_{A}^{S}$ values showed that the linkage disequilibrium in each group was significantly greater than zero (Table 1), indicating that the Japanese and European isolates could have diverged from different most recent common ancestors.

**TABLE 1:** Linkage disequilibrium for single nucleotide polymorphism (SNPs) from *Salmonella* Chester isolated in the Kanto region of Japan or Europe.

Group	*V*_*D*_	*V*_*e*_	$I_{A}^{S}$	Monte Carlo simulation		
				
				*Var* (*V*_*D*_)	*P*	*L*
Kanto region isolates	31.1481	4.7178	0.0162	0.5353	<0.001	6.0092
European isolates	209.4261	18.0041	0.0307	4.5976	<0.001	21.8087

### Different Genetic Traits Between the Japanese and European Isolates

**FIGURE 3:**
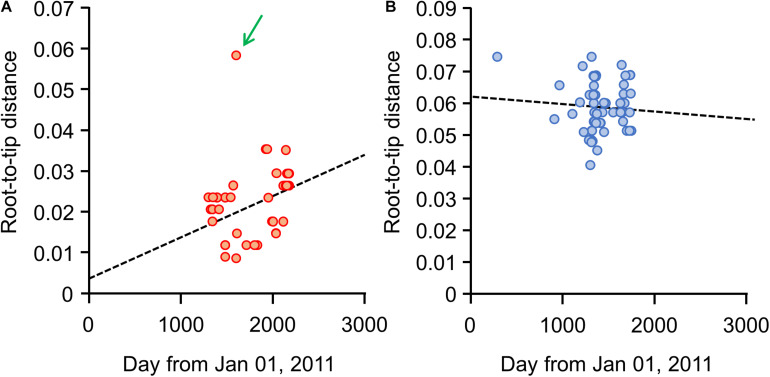
Root-to-tip analysis for each *S*. Chester isolated in the Kanto region of Japan or Europe. The X-axis represents the isolation date of each *S*. Chester isolate as the days lapsed from January 01, 2011. The Y-axis represents the genetic distance to each isolate from the root in the dendrogram for isolates from **(A)** the Kanto region or **(B)** from Europe. Dotted lines represent the regression lines drawn from **(A)** the Kanto region’s isolates, excluding isolate 29149, and **(B)** the European isolates. A green arrow indicates isolate 29149.

Our root-to-tip analysis revealed a different trend in the diversity change between the Japanese and European isolates. A positive correlation was observed between diversity in the Japanese isolates and their isolation dates, with the exception of isolate 29149 (Figure 3A), indicating that genetic diversity in the Japanese *S*. Chester isolates (excepting isolate 29149) increased as time advanced after the isolates originated from a single clone. In contrast, no such tendency was found when the European isolates were analyzed (Figure 3B).

**FIGURE 4:**
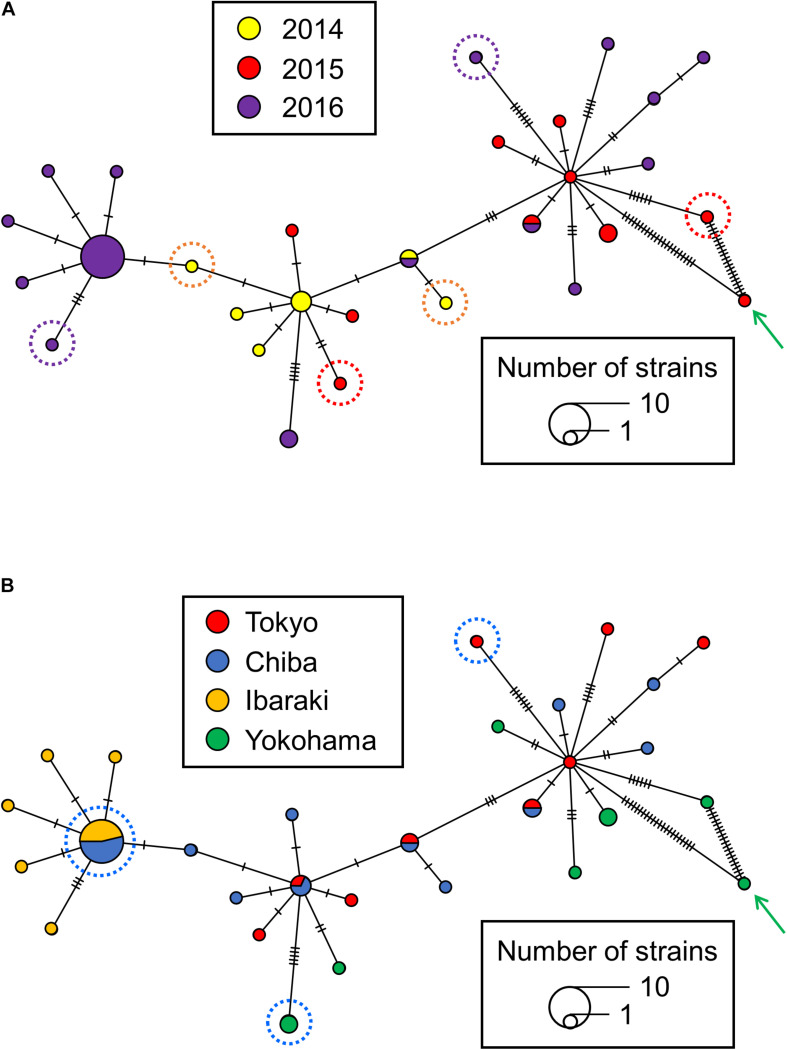
A minimum spanning tree (MST) reconstructed from the single nucleotide polymorphism (SNPs) from *S*. Chester isolated in the Kanto region of Japan. The colors of the nodes in panels **(A,B)** indicate the years and areas in which the *S*. Chester stains were isolated, respectively. **(A)** The isolates surrounded by orange, red and purple dotted lines show the maximum pair-wise distances between two isolates in 2014, 2015, and 2016, respectively. **(B)** The isolates surrounded by blue dotted lines show the maximum pair-wise distances of the isolates from Tokyo and Chiba and those of the Tokyo and Yokohama isolates. A green arrow indicates isolate 29149. A crossing bar on a connecting line indicates one SNP.

The MST analysis also showed that chronological variation accumulated in the Japanese isolates. Because isolate 29149 was set apart from the more clustered positions of the other isolates in the root-to-tip analysis (Figure 3A), it was excluded so as to evaluate the maximum pair-wise distances in the MST (Figures 4A,B). The maximum pair-wise distances between two isolates in 2014, 2015, and 2016 were 3, 12, and 16, respectively (Figure 4A). In contrast, no correlation was observed between genetic distance and geographic distance in the Japanese isolates. The maximum pair-wise distance for the isolates from Tokyo and Chiba was 11, while that of Tokyo and Yokohama was 16 (Figure 4B), although the geographic distances between Tokyo-Chiba and Tokyo-Yokohama (distances between the local authorities’ offices) were 39 and 27 km, respectively.

The *d*_*N*_/*d*_*S*_ ratios of the sequences from the Japanese and European isolates were 1.89 and 0.94, respectively. These values indicate that the Japanese isolates have been subjected to positive selection pressure, and that the European isolates have been subjected to negative selection pressure. While positive selection pressure causes individuals in a population to accumulate mutations, negative selection pressure excludes individuals with mutations that differ from the population (Tanner and Kingsley, 2018). No SNPs were located in the mismatch repair system genes (*mut*S, *mut*L, *mut*H, and *uvr*D) (Supplementary Table 3).

## Discussion

Our results refute the hypothesis that the temporary increase in *S*. Chester infections in the Kanto region of Japan was related to the *S*. Chester isolates spreading continuously in Morocco. The cluster and Bayesian cluster analyses showed that the Japanese and European isolates fell into two different clusters; thus, it is possible that the Japanese and European isolates are genetically distinct populations. Therefore, population genetic analysis was performed using *Φ_*PT*_* and $I_{A}^{S}$ values to confirm the genetic difference between the Japanese and European isolates in the two clusters. These values revealed that the Japanese and European isolates were genetically different from each other and were derived from each of the most recent ancestors. Fonteneau et al. (2017) suggested that *S*. Chester isolates belonging to the same clonal group had spread continuously in Morocco and that the spreading isolates (i.e., the European isolates) have caused outbreaks in Europe via Moroccan seafood. Therefore, it is likely that the temporary increase of *S*. Chester infections in Japan was caused by an infectious source other than the seafood imported from Morocco.

Our study has revealed that the *S*. Chester isolates originating from one clone emerged in the Kanto region via contaminated food items, which differed from the infection source of the European outbreaks. In general, when bacterial strains emerge via person-to-person contact, genetic distances in the strains are positively correlated with the geographical distances among the places where the strains are isolated (Brown et al., 2019). Conversely, bacterial strains emerging via the food supply chain lack this correlation. In our study, the MST for the Japanese isolates shows no correlation between the genetic and geographical distances in the isolates, suggesting that *S*. Chester emerged in the Kanto region via the food supply chain but not by person-to-person contact. Generally, the emergence of a foodborne pathogen requires two factors. The first factor is the introduction of a pathogen into a new host (Morse, 1995; Whittam et al., 1998). Newly emerging foodborne pathogens in the environment or in hosts outside of the human food supply chain would be introduced into a new host in the human food supply chain. However, there is no evidence that the Japanese isolates were introduced into a new host via the food supply chain because *S*. Chester was not reported in livestock and food samples by the national surveillance systems during its new emergence in the Kanto region between 2014 and 2016 (Ministry of Agriculture, Forestry and Fisheries, 2018; National Veterinary and Assay Laboratory, 2020)^5^^6^. The second factor relating to new emergence is the acquisition of genetic variations (Whittam et al., 1998). The root-to-tip analysis showed that the Japanese isolates originating from one clone accumulated mutations over time, suggesting that they can be defined as one strain and that this strain met the second factor’s requirement. However, it seems likely that the newly emerging *S*. Chester isolates were unable to establish themselves and adapt to their new environment because none were isolated after 2017 in Japan at the point of writing this paper.

The results of our tests to detect selective pressure (the *d*_*N*_/*d*_*S*_ ratio) acting on the isolates may explain why the Japanese *S*. Chester strain was not isolated after 2017. In general, the population of bacterial clones in a reservoir of persistent infection is subject to negative selection pressure, and the pressure excludes some clones with mutations that differ from the population (Tanner and Kingsley, 2018). Conversely, when a population of bacterial clones is subject to positive selection pressure outside the reservoirs, the pressure causes the clones to accumulate mutations (i.e., the diversity of the population increases). The *d*_*N*_/*d*_*S*_ ratio for the Japanese strain was >1, indicating that the genes possessed by these isolates have been subjected to positive selection (Stukenbrock and Bataillon, 2012). Therefore, the emerging Japanese strain has been under positive selection pressure, suggesting that it existed outside of any reservoir during its emergence. As to why *S*. Chester was not isolated in the Kanto region after 2017, the organism would have been unable to find suitable reservoirs in which to survive in the human food supply chain. In contrast, the *d*_*N*_/*d*_*S*_ ratio for the European isolates was <1, indicating that the genes in these isolates have been subjected to negative selection (Stukenbrock and Bataillon, 2012). Our root-to-tip analysis showed that genetic diversity in the European isolates has decreased over time (Figure 3B). These results suggest that the European isolates have already adapted to the reservoirs existing in the European human food supply chain. Fonteneau et al. (2017) also raised the possibility of persistent contamination sources in Morocco. Thus, the different fate of the Japanese strain and the European isolates may have been determined by their colonizing opportunities toward a suitable host.

We chose not to exclude isolate 29149 from Yokohama City from our analysis of the Japanese isolates when we evaluated the selective pressure acting on the isolates from Kanto. However, the root-to-tip analysis revealed that isolate 29149 was set apart from the other isolates in its position in this analysis, although the other positions were positively correlated. Similarly, the MST analysis showed that isolate 29149 was distant from the other isolates. These results indicate that isolate 29149 accumulated mutations faster than the other Japanese isolates did. Nevertheless, mutations in the mismatch repair genes encoding *MutS*, *MutL*, *MutH*, and *UvrD* proteins did not contribute to this phenomenon, despite these genes being the most critical factors leading to hypermutability in bacteria (Sheng et al., 2020), because no SNPs were identified in these genes in the WGS data for isolate 29149. One possible explanation for this may be that the additional genetic changes were induced by antibiotic use and bacteriological examination procedures. In antibiotic treatment of clinical cases, bacteria respond to antibiotic stress resulting in mutations occurring in the bacterial genome (Levert et al., 2010). Subculturing bacteria during their examination and detection can also cause genome variation (Iguchi et al., 2006; Yokoyama et al., 2017). However, we have no information with which to elucidate the influence of such factors. In this study, the *d*_*N*_/*d*_*S*_ ratio of the Japanese isolates other than isolate 29149 was 1.92. Therefore, even if isolate 29149 had been excluded from this study, this would not have changed our conclusion that the genes examined in the Japanese strain have undergone positive selection.

A limitation of the present study is that the WGS data from the Japanese *S*. Chester isolates could not be compared with the data from the isolates collected in Japan before 2013. Our data revealed that the Japanese isolates emerging during 2014–2016 originated from one clone. It is possible that an *S*. Chester clone became established in its host in the Japanese environment and the clone emerging from this time may have arisen from the pre-existing clone. This possibility, however, seems unlikely. In the first place, infections with *S*. Chester have been rare among all the reported NTS infections in Japan (Katoh et al., 2015). If the emerging *S*. Chester clone had been present in the Japanese environment, similar emergences of the *S*. Chester isolates investigated in this study would have occurred. There was only one large *S*. Chester outbreak in 1998. However, the isolates from 1998 were classified as belonging to ST343 using MLST. In the present study, the Japanese strains all belonged to ST1954. Therefore, the genetic distance between our Japanese isolates and the isolates from 1998 was too large to evaluate their genetic relationships. Even if the *S*. Chester isolates isolated before 2013 were to be collected from all the local public health institutes throughout Japan and the WGS data were compared between our Japanese isolates and the pre-2013 collected isolates, it is unlikely that the comparison would provide new insights into the findings of this study.

## Conclusion

We conclude that the *S*. Chester isolates originating from one clone emerged in Japan’s Kanto region via contaminated foods other than imported Moroccan seafood. However, the emerging isolates could not establish any reservoirs for survival in the human food supply chain in Japan, and *S*. Chester was not isolated in the Kanto region after 2017.

## Data Availability Statement

The datasets presented in this study can be found in online repositories. The names of the repository/repositories and accession number(s) can be found in the article/Supplementary Material.

## Author Contributions

NA, EY, KM, and SH conceptualized and designed this study. NA, EY, YA, NK, YM, and SF isolated *S*. Chester from human stools, and identified the isolates as *S*. Chester. TS, YM, NJ-D, SL, KM, and MK performed WGS on the *S*. Chester isolates. TS, NJ-D, SL, and MK processed and analyzed the WGS data. NA, TS, EY, MK, and SH performed molecular epidemiological and population genetic analyses using the SNPs extracted from the WGS data. KI, KN, MS and HK assisted with project development and data analysis. NA, EY, KM, and SH wrote the draft manuscript. All authors have commented on and agreed upon the final manuscript.

## Conflict of Interest

The authors declare that the research was conducted in the absence of any commercial or financial relationships that could be construed as a potential conflict of interest.

## Publisher’s Note

All claims expressed in this article are solely those of the authors and do not necessarily represent those of their affiliated organizations, or those of the publisher, the editors and the reviewers. Any product that may be evaluated in this article, or claim that may be made by its manufacturer, is not guaranteed or endorsed by the publisher.

## Footnotes

1 https://www.who.int/news-room/fact-sheets/detail/salmonella-(non-typhoidal)

2
http://idsc.tokyo-eiken.go.jp/epid/

3
https://www.maff.go.jp/j/tokei/kouhyou/kokusai/houkoku_gaikyou.html

4
http://www.genomicepidemiology.org/

5
https://www.maff.go.jp/j/syouan/nouan/kome/k_yasai/

6
https://www.maff.go.jp/nval/yakuzai/yakuzai_p3.html
