# Supplementary material for: Whole Genome Analysis Detects the Emergence of a Single Salmonella enterica Serovar Chester Clone in Japan’s Kanto Region
Source: Front Microbiol. 2021 Jul 27;12:705679. doi: 10.3389/fmicb.2021.705679 (PMC8354586; doi:10.3389/fmicb.2021.705679)
Supplement: Supplementary file 2 [file Presentation_1.PPTX]

## Slide 1
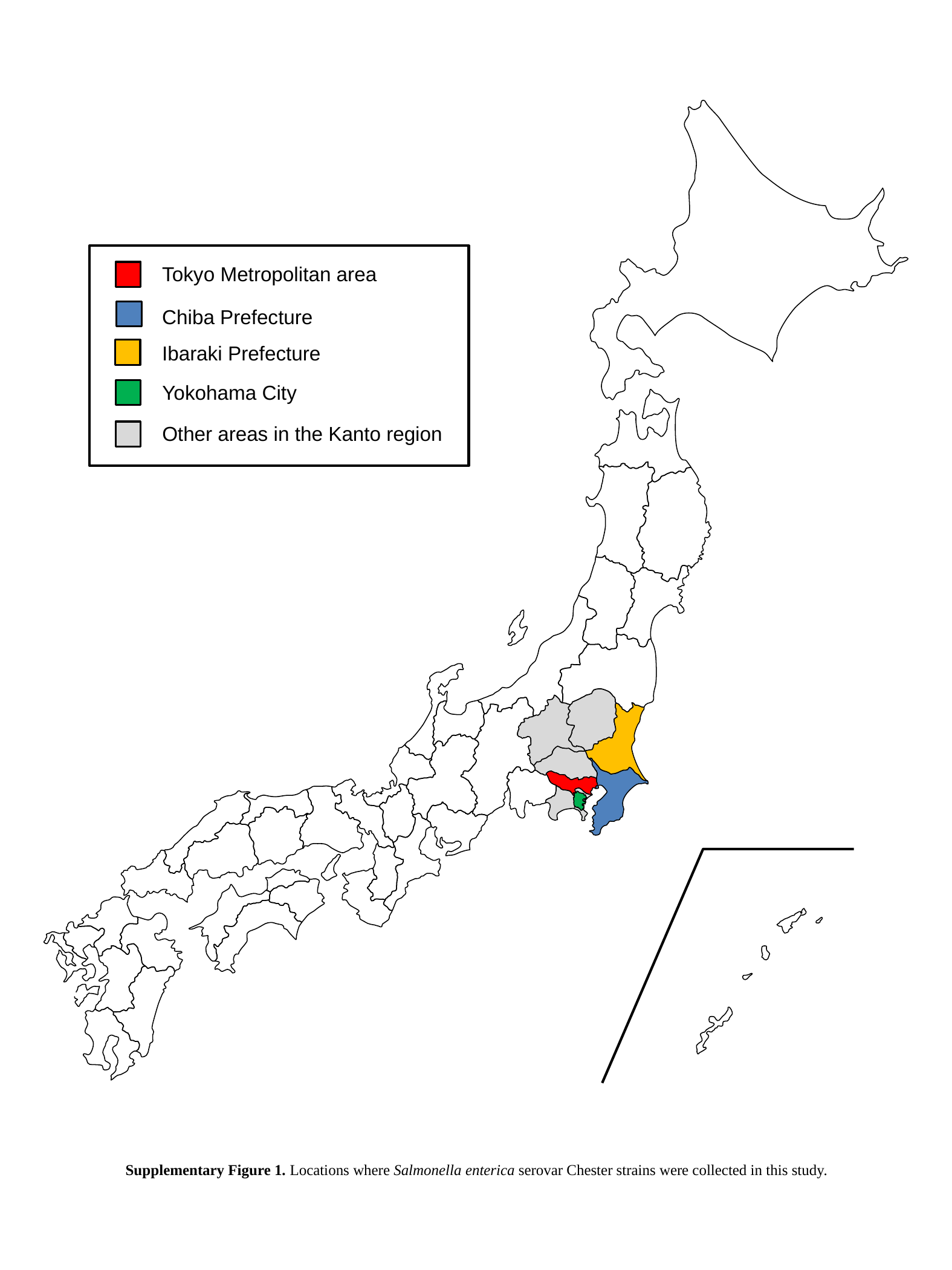

Tokyo Metropolitan area
Chiba Prefecture
Ibaraki Prefecture
Yokohama City
Other areas in the Kanto region
Supplementary Figure 1. Locations where Salmonella enterica serovar Chester strains were collected in this study.
